# Medical Management of Modifiable Risks: Improving Survival in High-Risk Prostate Cancer Patients Receiving Brachytherapy

**DOI:** 10.3390/jcm15145414

**Published:** 2026-07-10

**Authors:** Shalini Moningi, Grgur Mirić, Robert W. Galbreath, Ryan Fiano, Kent E. Wallner, Mutlay Sayan, Peter F. Orio, Martin King

**Affiliations:** 1 Department of Radiation Oncology, Taussig Cancer Institute, Cleveland Clinic Foundation, 9500 Euclide Avenue, CA-50, Cleveland, OH 44195, USA; 2 Urologic Research Institute, Sarasota, FL 34236, USA; 3 Department of Exercise Physiology, Ohio University Eastern, St. Clairsville, OH 43950, USA; 4 Department of Radiation Oncology, University of Washington, Seattle, WA 98195, USA; 5 Department of Radiation Oncology, Brigham and Women’s Hospital, Boston, MA 02115, USA; 6 Dana Farber Cancer Institute, Boston, MA 02215, USA; peter_orio@dfci.harvard.edu

**Keywords:** prostate cancer, brachytherapy, Pd-103, high risk, medical comorbidities

## Abstract

**Background:** High-risk (HR) prostate cancer has a propensity for local and distant progression with ultimate death, mandating aggressive locoregional and systemic treatment approaches to maximize oncologic outcomes. Although brachytherapy (BT) with supplemental therapies has demonstrated favorable biochemical and quality of life outcomes, improvements in overall survival have been hampered by an excessive incidence of non- prostate cancer deaths. In this HR study, we report on biochemical failure (BF), prostate cancer-specific mortality (PCSM), overall mortality (OM) and patterns of death with recommendations for the mitigation of non-prostate cancer deaths. **Materials and Methods:** From April 1995 to November 2018, 577 consecutive HR patients were treated with LDR BT (97.9% Pd-103). Patients were stratified into three age cohorts: ≤ 59, 60–69 and ≥70 years. The BT prescription dose was prescribed to the prostate gland with generous peri-prostatic margins and the proximal 10mm of the seminal vesicles. 94.6% received supplemental EBRT (45–50.4 Gy) and 63.3% received androgen deprivation therapy (ADT) (median duration 12 months). Post-implant CT-based dosimetry was performed on day 0. BF was defined as a PSA > 0.40 ng/mL after nadir. The cause of death was determined for each patient. Patients with metastatic prostate cancer or non-metastatic castrate resistant prostate cancer who died of any cause were classified as dead of prostate cancer. All other deaths were attributed to the immediate cause. Multiple clinical, pathologic and treatment were evaluated for impact on patient outcomes. **Results:** Of the patients, 87.5% (median follow-up 8.9 years) presented with a single HR factor. The day 0 D90 was 122.5%. Overall, the 15-year BF, PCSM and OM were 12.4%, 5.6% and 51.7%. When stratified by age, there was no significant difference in BF or PCSM. The median post- treatment PSA in biochemically controlled patients was <0.01 ng/mL. In all three cohorts, OM increased linearly for the first 10 years and then approximately doubled from years 10 to 15. Moreover, 239 patients died: 10.9% due to prostate cancer, 38.1% from cardiovascular (CV) disease and 28.4% from other malignancies (to include one rectal and three bladder cancers). In MVA, BF was most closely related to percent positive biopsies (*p* < 0.001, SHR 1.018), PCSM to Gleason score (*p* = 0.004, SHR 2.884) and percent positive biopsies (*p* = 0.005, SHR 1.021) and OM to age (*p* < 0.001, HR 1.075) and tobacco (*p* < 0.001, HR 2.374). **Conclusions:** Despite high cancer control rates, overall survival was limited by a preponderance of CV and non-prostate cancer deaths, which were 6 times more likely than prostate cancer deaths. The implementation of a comprehensive multidisciplinary survivorship program will be essential to impact longevity in this patient population.

## 1. Introduction

Prostate cancer is the most common cancer diagnosis in men in North America. In 2021, an estimated 3.3 million men were living with prostate cancer in the United States [1]. The optimal management of prostate cancer remains controversial without clear consensus for any risk group. This is especially true for high-risk (Gleason Grade Group 4–5, PSA > 20 ng/mL or clinical stage T3) prostate cancer, and it is relevant because of its attendant risk of local and distant progression with ultimate death if not successfully treated.

Over the past decade, radical prostatectomy (RP) has been increasingly utilized for high-risk patients with biochemical control rates of 40–50% commonly reported [2]. Higher rates of biochemical control and fewer distant metastases have been reported for external beam radiation therapy (EBRT) combined with androgen deprivation therapy (ADT) compared to EBRT alone [3]. Because of intraprostatic dose escalation and the ability to treat generous peri-prostatic margins, brachytherapy is an attractive alternative [4]. Multiple HR brachytherapy studies (usually in combination with supplemental therapies) have reported improved outcomes [5,6]. Following combined-modality brachytherapy, a multi-institutional trial of Gleason score 9–10, patients reported fewer biochemical failures, reductions in distant metastases and prostate cancer deaths, and improved overall survival in comparison to radical prostatectomy +/− radiotherapy or EBRT + ADT [6].

Patient age is another point of contention in treatment selection criteria. Despite favorable biochemical and functional outcomes in younger patients, a bias remains for surgical expiration in this cohort. In contrast, because of concerns of competing causes of death to include cardiovascular, non-prostate cancer malignancies and degenerative etiologies, older patients are often deprived of potentially curative approaches. Even when controlled for Gleason score and PSA, older patients are more likely to experience progression. Biologic differences in prostate cancer aggressiveness appear to be inversely related to patient age [7]. In fact, greater than 50% of all prostate cancer deaths occur in men in their 80s [7].

In this study, we assessed the long-term oncologic and survival outcomes (stratified by age) in a large single-institution cohort of HR prostate cancer patients treated with low-dose rate (LDR) brachytherapy with the goal to identify opportunities to improve survivorship and long-term health.

## 2. Materials and Methods

From April 1995 through to November 2018, 577 consecutive patients with high-risk prostate cancer (Gleason Grade Group 4–5, PSA > 20 ng/mL or clinical stage T3) underwent low-dose rate brachytherapy (97.9% Pd-103 and 2.1% I-125). Brachytherapy was recommended for HR patients with a life expectancy of at least 5 years and adequate urinary function to include a post-void residual of <100 cc (without medication). Patients were clinically staged using medical history, physical examination including digital rectal examination, serum PSA, bone scans and computed tomography of the chest/abdomen/pelvis. Medical comorbidities were assessed by review of medical records, discussions with treating physicians and patient interviews. Beginning in 2002, serum testosterone determinations were added to the pre-treatment evaluation. This retrospective study met all ethical, scientific and administrative standards mandated by our IRB for research involving human subjects. The study was conducted with IRB approval (S24–25).

### 2.1. Brachytherapy Planning

The brachytherapy planning target volume consisted of the prostate gland with 5 mm. peri-prostatic margins except posteriorly (0–2 mm margin) and the proximal 1.0 cm of the seminal vesicles [8]. This resulted in a planning target volume approximately 1.9 times the actual prostate volume. All post-implant dosimetric calculations were based on day 0 CT evaluation. Prior to 2000, a preplanned approach was utilized. After that date, the preplan served as a template for intraoperative dosimetry with the goal to obtain a post-implant day 0 target volume D90 of approximately 120% and a mean urethral dose of about 110% of prescription. Implant boost doses were 90 Gy for Pd-103 and 110 Gy for I-125. For monotherapy, the prescription dose for Pd-103 was 90 Gy. All brachytherapy procedures were performed by a single brachytherapist (GM).

### 2.2. External Beam Radiation

Supplemental external beam radiation therapy (EBRT) (45–50.4 Gy in 1.8–2.0 Gy fractions) was administered prior to the brachytherapy boost. The EBRT target volume consisted of the prostate gland and the seminal vesicles with margin and the pelvic lymph nodes. Initially, patients were treated with a 6-field 3-dimensional conformal technique. After 2005, all patients were treated with 5-field intensity modulated radiation therapy (IMRT). Patients underwent brachytherapy boost 4 to 15 days following the completion of EBRT. All patients received supplemental EBRT except those who refused or had previously received pelvic radiation. Overall, 94.6% of patients received pelvic EBRT.

### 2.3. Androgen Deprivation Therapy

When prescribed, ADT was initiated 3 months prior to brachytherapy and consisted of either an LHRH agonist with an antiandrogen or an LHRH antagonist. ADT was used for size reduction and/or adverse pathologic features at the discretion of the treating physician. Median ADT duration was 4 months and 12 months in the ≤6 month and >6 month duration cohorts (maximum duration 36 months).

### 2.4. Outcomes

Primary outcome measures were biochemical failure (BF), prostate cancer-specific mortality (PCSM), and overall mortality (OM). Patients were monitored by physical examination, including digital rectal examination and serum PSA and testosterone determinations at 3- and 6-month intervals. Biochemical failure was defined as a PSA > 0.40 ng/mL after nadir. The cause of death was determined for each deceased patient. Patients with metastatic prostate cancer or hormone refractory disease without obvious metastases who died of any cause were classified as dead of prostate cancer. All other deaths were attributed to the immediate cause of death. The cause of death was determined by review of departmental, hospital and primary care records and death certificates. Patients and/or their family were contacted at least every 6 months until death. Differences in cause of death were stratified by age at implant.

### 2.5. Statistical Analysis

Clinical, treatment and dosimetric values that were collected as continuous variables were compared using a one-way ANOVA. Variables collected as categorical were presented using contingency tables. Pearson’s Chi-square was used to determine the significance of the association across the age groups. Overall mortalities were compared across the 3 age groups using Cox regression analysis. Univariate analyses were used to determine the hazard ratios of select variables with overall mortality. Variables with *p*-values ≤ 0.10 were entered into a multivariate analysis. The rate of BF and PCSM for the different age groups were determined using Fine-Gray competing risk analysis and presented using cumulative incidences. Univariate analyses were used to determine the subdistribution hazard ratios of select variables with BF and PCSM. Variables that provided *p*-values ≤ 0.10 were then entered into a multivariate analysis. In addition, the Fine-Gray model was used to compare non-cancer deaths across the 3 age groups. STATA version 18.0 software (StataCorp, College Station, TX, USA) and SPSS version 29.0 (Chicago, IL, USA) were used for analysis with significance set at *p* < 0.05.

## 3. Results

**Figure 1:**
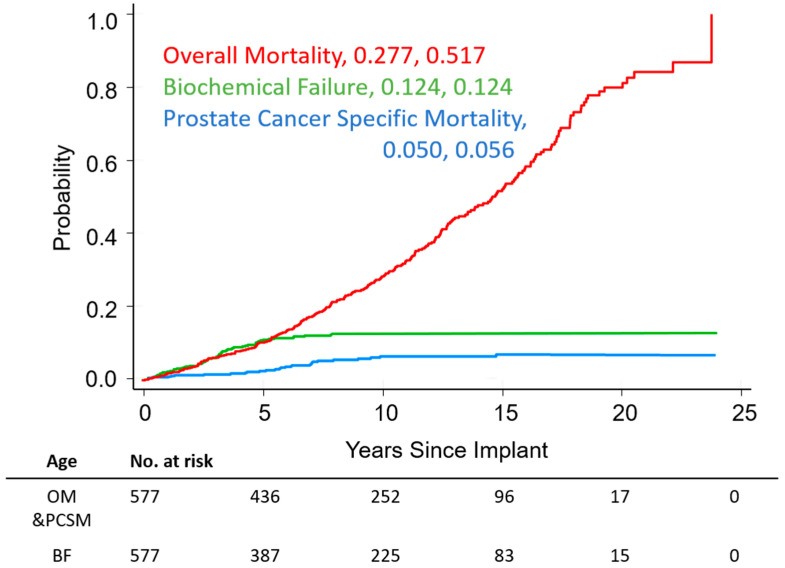
Representation of the 10 and 15 year overall mortality, prostate cancer-specific mortality and biochemical failure for high-risk patients.

Figure 1 summarizes the clinical, treatment and dosimetric parameters of the 577 consecutive patients who comprised this study, stratified by patient age. For the entire group, the mean and median follow-up was 9.4 and 8.9 years, respectively. Of the patients, 87.5% presented with only one high-risk factor. This finding was most prominent in the two older age groups (*p* = 0.012). Patients ≤ 59 years of age presented with a statistically higher PSA (*p* < 0.001), smaller prostate glands (*p* < 0.001), greater percent positive biopsies (*p* = 0.040) and were more likely to currently use tobacco (*p* <0.001). The youngest cohort was statistically least likely to present with diabetes mellitus, coronary artery disease or hypercholesteremia. There was no statistically significant difference in Gleason Group, the use/duration of ADT, utilization of EBRT or baseline testosterone among the 3 cohorts. ADT was administered to 63.3% of patients for a median duration of 12 months in those patients receiving ADT. Moreover, 97.9% of patients were implanted with Pd-103. Although there were statistically significant differences in day 0 dosimetry, the differences were not clinically meaningful and represented significant dose escalation. The day 0 D90 was >120.5% for all three age groups. For all biochemically controlled patients, the median most recent PSA was <0.01 ng/mL.

**Figure 2:**
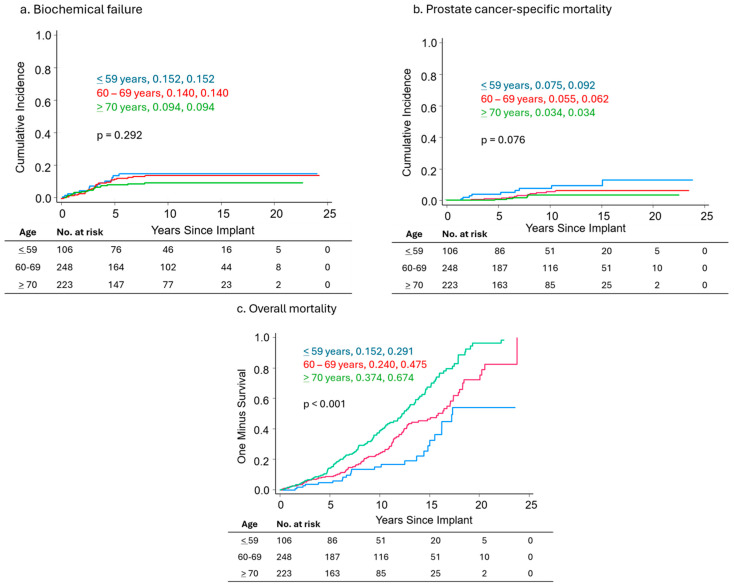
Representation of the 10 and 15 year biochemical failure (**a**), prostate cancer-specific mortality (**b**), and overall mortality (**c**) of high-risk patients, stratified by age.

**Figure 3:**
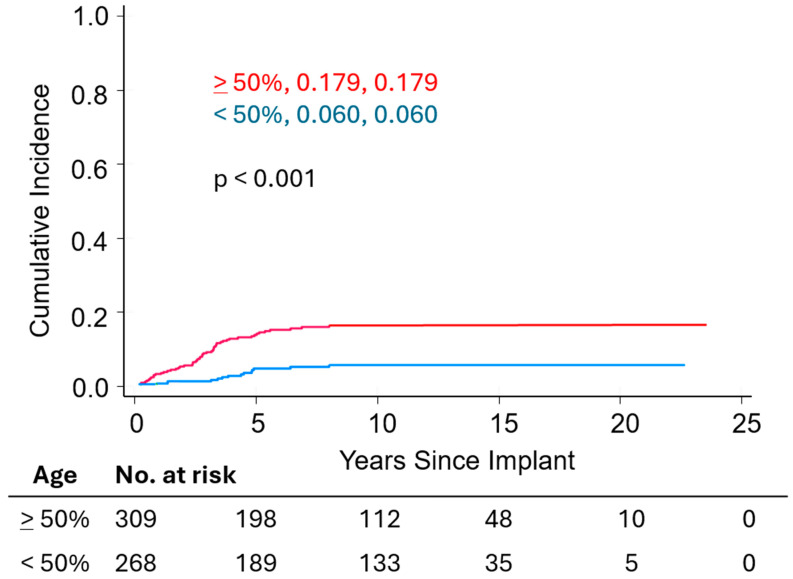
Representation of the 10 and 15 year biochemical failure of high-risk patients, stratified by percent positive biopsy groups.

**Figure 4:**
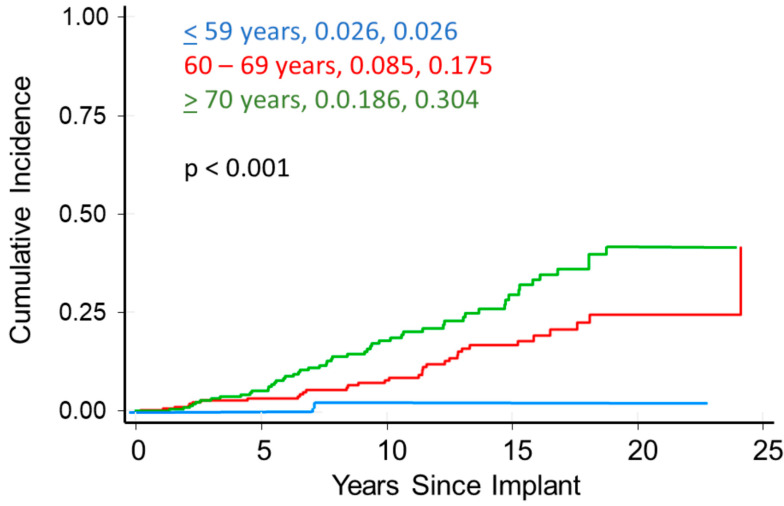
Representation of the 10 and 15 year cardiovascular-related mortality, stratified by age.

Figure 1 illustrates the 10- and 15-year BF, PCSM and OM for the entire group of 577 patients. Adverse BF and PCSM events stabilized prior to year 10 with minimal changes through year 15. In contrast, OM increased linearly through year 10 and then virtually doubled by year 15. Figure 2 stratifies outcomes by age. There were no statistically significant differences in BF (*p* = 0.292) or PCSM (*p* = 0.092), but as expected age significantly impacted OM (*p* < 0.001). BF was closely related to percent positive biopsies (PPB) with 15-year BF rates of 17.9% and 6.0% for ≥50% and <50% (Figure 3, *p* < 0.001). Separating PPB into four groups further emphasized the influence of PPB on oncologic control (<25%, 25–49.9%, 50–74.9% and ≥75% with BF of 3.5%, 7.6%, 15.5% and 20.5%; *p* < 0.001). When stratified by age, cardiovascular disease predicted for overall mortality (Figure 4).

There were 239 deaths recorded in this patient population. Prostate cancer accounted for 26 deaths (10.9% of all deaths), while 91 patients died of cardiovascular disease (38.1%) and 68 died of other malignancies (28.4%). The majority of these other cancer deaths were tobacco-related. One patient died of rectal cancer and 3 of bladder cancer. Non-cancer-specific mortality was closely related to patient age (≤59 vs. 60–69, *p* = 0.006, SHR = 7.514; ≤59 vs. ≥70, *p* < 0.001, SHR = 13.789).

In Fine–Gray analysis, BF was most closely related to percent positive biopsies (SHR: 1.018; 95% CI = 1.008–1.028; *p* < 0.001) (Supplemental Table S1a). PCSM was impacted by Gleason Group (SHR: 2.884; 95% CI = 1.417–5.871; *p* = 0.004) and percent positive biopsies (SHR: 1.021; 95% CI = 1.006–1.036; *p* = 0.005) (Supplemental Table S1b). In Cox regression analysis, OM was most closely related to age (HR: 1.075; 95% CI = 1.053–1.098; *p* < 0.001) and tobacco (HR: 2.374; 95% CI = 1.587–3.550; *p* < 0.001) (Supplemental Table S1c). Stratification into year of treatment (pre-/post-2005), the use or duration of ADT or comorbidities at presentation did not impact BF, PCSM or OM. An interaction between ADT and CV risk on OM was not discerned (*p* = 0.684). An analysis of ADT and CV risk on PCSM was not feasible because of a lack of events.

## 4. Discussion

In this single-institution cohort of 577 consecutive patients with high-risk prostate cancer, we report outstanding long-term oncologic outcomes with 15- year BF and PCSM of 12.4% and 5.6%, respectively. BF and PCSM outcomes were consistent across the three evaluated age groups without statistically significant differences. However, overall mortality nearly doubled from 27.7% at 10 years to 51.7% at 15 years with more pronounced increases in older patients. The majority of the deaths seen in this cohort were due to non-prostate cancer causes, mostly from either cardiovascular disease or non-prostate cancer malignancy. Tobacco use was the strongest predictor of overall mortality (Table S1c). The prevalence of cardiovascular and tobacco-related cancer deaths lead us to strongly support the development of survivorship programs as a framework to mitigate the adverse consequences from these two modifiable health risks.

Our current results further add to the data on the efficacy of brachytherapy in the HR population and complement previously reported prospective and retrospective studies [4,5,6]. The very low rates of BF and PCSM in our study are in part due to a relatively favorable clinical presentation upfront (87.5% presented with only one high risk factor), brachytherapy’s ability (especially Pd-103) to allow for the escalation of intraprostatic doses and to aggressively treat extracapsular disease, including the proximal seminal vesicles. In the current study, the median most recent PSA was <0.01 ng/mL in biochemically controlled patients (Table 1). These results are consistent with randomized trial data that demonstrated that monotherapeutic brachytherapy delivers a high biologic effective dose (BED) to the prostate and periprostatic regions with resultant low rates of biochemical failure with undetectable post-treatment PSA in those patients [4].

Although advancing age has been correlated with a poor prognosis, investigators have reported favorable oncologic outcomes when older patients receive aggressive treatment [9,10]. The findings from our study also support this finding. We found no statistical differences in BF or PCSM among the three cohorts (Figure 2).

In this study, the modifiable health risks of cardiovascular disease and tobacco consumption resulted in a significant number of non-prostate cancer deaths with deaths from these causes being six times more likely than prostate cancer. Although our study did not implicate ADT in overall mortality (Supplemental Table S1c), data from multiple clinical trials support increased death from ADT use [11,12,13,14]. The identification of patients with CV disease is important because CV disease is treatable and treatment decreases mortality. Although currently there are no specific recommendations from the NCCN to mitigate cardiac risk factors in men with prostate cancer, we suggest that workup should include an assessment of cardiovascular status and the comprehensive treatment of all comorbidities including hypertension, diabetes mellitus, hypercholesterolemia/hyperlipidemia and obesity. A heart healthy diet along with routine exercise and tobacco cessation should also be encouraged. Multiple studies have demonstrated the value of aerobic exercise and resistance training in reducing prostate cancer progression and death. Additionally, 150 min of exercise/week has been reported to decrease overall mortality and prostate cancer deaths [15]. In addition, tobacco cessation decreases cardiovascular risk, cancer risk and death [16].

Strengths of our study include a relatively large sample size with all patients treated and followed by a single physician, uniformity of treatment delivery with a consistent brachytherapy and EBRT philosophy, and follow-up until death. The limitations of our study include the retrospective analysis of a single institution experience, the absence of data regarding the duration and severity of cardiovascular disease and other comorbidities, ADT delivered in a non-controlled setting and changes in survivorship care over time. These shortcomings may affect the generalizability of our results.

In summary, despite improved biochemical efficacy for all prostate cancer treatment modalities, improvements in overall survival remain elusive. Increasing overall survival will require prostate cancer physicians to emphasize survivorship issues to include the medical management of comorbidities, lifestyle changes and the development of multidisciplinary survivorship programs. In summary, we recommend primary care and cardiology (when appropriate) management for assessment and treatment of medical comorbidities, daily aerobic exercise, resistance training 3 times/week, a heart healthy diet, cessation of all tobacco products and adherence to preventative screening procedures, such as colonoscopy, DEXA scans and CT chest (in long-term smokers) [17].

## 5. Conclusions

Despite high cancer control rates, the overall survival was limited by a preponderance of CV and non-prostate cancer deaths, which were 6 times more likely than prostate cancer deaths. The development and implementation of a comprehensive, multidisciplinary survivorship program is essential to improving longevity in this patient population.

## Figures and Tables

**Table 1:** Clinical, treatment and dosimetric parameters.

	≤59 Years n = 107		60–69 Years n = 248		≥70 Years n = 223			All Patients n = 578	
**Variable**	**Mean (SD)**	**Med.**	**Mean (SD)**	**Med.**	**Mean (SD)**	**Med.**	**p ^a^**	**Mean (SD)**	**Med.**
Age (yrs)	55.0 (3.4)	56.0	64.6 (2.9)	65.0	73.6 (2.9)	73.0	<0.001	66.3 (7.4)	67.0
Follow up (yrs)	10.1 (5.3)	9.7	9.6 (5.5)	9.3	8.9 (4.7)	8.7	0.083	9.4 (5.2)	8.9
Pretreatment PSA (ng/m^2^)	19.1 (20.0)	12.2	13.8 (14.0)	8.4	12.3 (10.2)	8.3	<0.001	14.2 (14.2)	8.8
Prostate vol. (cm^3^)	20.5 (6.1)	19.0	25.4 (9.5)	23.7	27.7 (10.5)	25.2	<0.001	25.4 (9.7)	23.4
Body mass index	29.5 (5.6)	28.0	29.5 (4.9)	29.2	28.7 (4.5)	27.9	0.072	29.2 (4.9)	28.4
Percent positive biopsy	57.8 (28.8)	56.3	49.6 (27.3)	49.0	51.5 (28.6)	50.0	0.040	51.9 (28.1)	50.0
Average urethral dose (%)	119.7 (11.0)	117.7	116.2 (13.5)	115.7	114.5 (13.6)	113.8	0.006	116.2 (13.2)	115.5
V100 (%vol)	98.2 (1.8)	98.7	97.5 (3.1)	98.4	96.9 (3.6)	98.1	0.002	97.4 (3.1)	98.3
V150 (%vol)	73.8 (8.8)	75.2	72.0 (10.1)	73.4	70.6 (11.4)	72.6	0.029	71.8 (10.4)	73.7
V200 (%vol)	44.9 (9.0)	45.9	43.3 (9.6)	43.9	42.3 (10.1)	43.3	0.069	43.2 (9.7)	44.1
D90 (% prescribed dose)	125.2 (10.2)	125.0	122.5 (11.4)	122.5	120.5 (12.1)	120.9	0.002	122.2 (11.6)	122.5
Most recent PSA (ng/m^2^)	0.06 (0.18)	<0.001	0.02 (0.05)	<0.001	0.01 (0.04)	<0.001	0.028	0.02 (0.10)	<0.001
	**Count**	**(%)**	**Count**	**(%)**	**Count**	**(%)**	**p ^x^**	**Count**	**(%)**
Clinical stage T1b-T2a	81	(76.4)	179	(72.2)	162	(72.6)	0.696	422	(73.1)
T2b-T2c	25	(23.6)	69	(27.8)	61	(27.4)		155	(26.9)
Gleason Group 1	5	(4.7)	9	(3.6)	6	(2.7)	0.549 ^f^	20	(3.5)
2	2	(1.9)	6	(2.4)	6	(2.7)		14	(2.4)
3	10	(9.4)	23	(9.3)	10	(4.5)		43	(7.5)
4	48	(45.3)	123	(49.6)	115	(51.6)		286	(49.6)
5	41	(38.37)	87	(35.1)	86	(38.6)		214	(37.1)
ADT duration 0	36	(34.0)	95	(38.3)	81	(36.3)	0.274 ^f^	212	(36.7)
≤6 mons.	5	(4.7)	27	(10.9)	22	(9.9)		54	(9.4)
≥6 mons.	65	(61.3)	126	(50.8)	120	(53.8)		311	(53.9)
Isotope Pd-103	103	(97.2)	245	(98.8)	217	(97.3)	0.432 ^f^	565	(97.9)
I-125	3	(2.8)	3	(1.2)	6	(2.7)		12	(2.1)
EBRT no	102	(96.2)	233	(94.0)	211	(94.6)	0.716 ^f^	546	(94.6)
Hypertension no	43	(40.6)	122	(49.2)	115	(51.6)	0.169	280	(48.5)
Diabetes no	4	(3.8)	41	(16.5)	26	(11.7)	0.002f	71	(12.3)
Coronary artery disease no	10	(9.4)	40	(16.1)	60	(26.9)	<0.001	110	(19.1)
Hypercholesterolemia no	23	(21.7)	83	(33.5)	79	(35.4)	0.032	185	(32.1)
Tobacco use never	40	(37.7)	86	(34.7)	86	(38.6)	<0.001	212	(36.7)
former	32	(30.2)	115	(46.4)	121	(54.3)		268	(46.4)
current	34	(32.1)	47	(19.0)	16	(7.2)		97	(16.8)
Testosterone ^t^ low/lower 1/3 norm	54	(67.5)	120	(72.7)	113	(71.1)	0.731	287	(71.0)
middle 1/3 norm	18	(22.5)	35	(21.2)	37	(23.3)		90	(22.3)
Upper 1/3 norm/high	8	(10.0)	10	(6.1)	9	(5.7)		27	(6.7)
Perinueral invasion no	51	(49.1)	111	(44.8)	109	(48.9)	0.648	271	(47.0)
Number of high risk factors 1	84	(79.2)	223	(89.9)	198	(88.8)	0.012 ^f^	505	(87.5)
2	19	(17.9)	24	(9.7)	25	(11.2)		68	(11.8)
3	3	(2.8)	1	(0.4)	0	(0.0)		4	(0.7)

^t^ Not all patients had testosterone values. ^a^ One-way ANOVA. ^f^ Fisher’s exact test. ^x^ Chi-square.

## Data Availability

All data are housed at Filemaker (a Google company) and Bethany College.

## Supplementary Materials

The following supporting information can be downloaded at: https://www.mdpi.com/article/10.3390/jcm15145414/s1, Table S1a: Univariate and multivariate competing risks analysis for biochemical failure; Table S1b: Univariate and multivariate competing risks analysis for prostate cancer-specific mortality; Table S1c: All-cause mortality univariate and multivariate analysis.
