# Supplementary material for: Medical Management of Modifiable Risks: Improving Survival in High-Risk Prostate Cancer Patients Receiving Brachytherapy
Source: J Clin Med. 2026 Jul 10;15(14):5414. doi: 10.3390/jcm15145414 (PMC13412678; doi:10.3390/jcm15145414)
Supplement: Supplementary file 1 [file jcm-15-05414-s001.zip › jcm-4307921-supplementary.pdf]

Supplementary data

Supplementary Table S1a. Univariate and multivariate competing risks analysis for biochemical failure.

	Biochemical Failure					
	Univariate			Multivariate		
**Variable**	**p**	**SHR**	**(CI)**	**p**	**SHR**	**(CI)**
Age	0.103	0.974	(0.943-1.005)			
Pre-implant PSA	0.088	1.013	(0.998-1.027)	0.322	1.007	(0.993-1.022)
Gleason group[g]	< 0.001	1.318	(1.004-1.729)	0.096	1.246	(0.962-1.615)
Body mass index	0.081	0.955	(0.977-1.006)	0. 214	0.968	(0.915-1.012)
Percent positive biopsies	< 0.001	1.022	(1.013-1.031)	< 0.001	1.018	(1.008-1.028)
Perineural Invasion no/yes	0.019	1.821	(1.105-3.000)	0.335	1.018	(0.765-2.194)
%D90	0.774	1.003	(0.981-1.026)			
EBRT[c] no/yes	0.965	1.026	(0.323-3.265)			
ADT[c] no/yes	0.778	0.930	(0.561-1.541)			
Post-2004 implant No/yes	0.069	0.635	(0.389-1.037)	0.105	0.663	(0.403-1.090)
ADT duration [a, c]	0.553					
none vs ≤ 6 months	0.294	0.564	(0.194-1.644)			
none vs > 6 months	0.963	0.988	(0.592-1.650)			
Hypertension[c] no/yes	0.192	0.726	(0.448-1.174)			
Tobacco[c]	0.494					
never vs former	0.264	0.742	(0.439-1.253)			
never vs current	0.439	0.750	(0.364-1.546)			
Diabetes melitus[c] no/yes	0.194	0.541	(0.215-1.366)			
Cardiovascular disease[c] no/yes	0.095	0.532	(0.253-1.116)	0.133	0.555	(0.257-1.197)
Hypercholesterolemia[c] no/yes	0.138	0.660	(0.381-1.144)			
No. high risk factors (1 base)[c, n]	0.002					
1 vs 2	0.008	2.214	(1.233-3.974)			
1 vs 3	0.012	8.475	(1.592-45.120)			

[a] ADT duration was not included in the multivariate analysis because it is directly related to ADT. [c]Categorical variable. [g] Gleason group was entered as a continuous variable. [n] count are already 5included in the multivariate analysis.

Supplementary Table S1b. Univariate and multivariate competing risks analysis for prostate cancer-specific mortality.

	Prostate Cancer Specific Mortality					
	Univariate			Multivariate		
**Variable**	**p**	**SHR**	**(CI)**	**p**	**SHR**	**(CI)**
Age	0.025	0.943	(0.896-0.993)	0.427	0.976	(0.919-1.037)
Pre-implant PSA	0.691	1.006	(0.976-1.037)			
Gleason group[g]	< 0.001	3.552	(1.755-7.188)	0.004	2.884	(1.417-5.871)
Body mass index	0.890	1.006	(0.929-1.089)			
Percent positive biopsies	< 0.001	1.029	(1.014-1.043)	0.005	1.021	(1.006-1.036)
Perineural Invasion no/yes	0.266	1.561	(0.712-3.421)			
%D90	0.998	1.000	(0.967-1.034)			
EBRT[c] no/yes	0.907	1.127	(0.153-8.279)			
Post-2004 implant No/yes	0.870	0.934	(0.414-2.107)			
ADT[c] no/yes	0.107	2.416	(0.826-7.065)			
ADT duration [a, c]	0.254					
none vs ≤ 6 months	0.369	2.005	(0.440-9.143)			
none vs > 6 months	0.099	2.490	(0.843-7.356)			
Hypertension[c] no/yes	0.108	0.526	(0.241-1.151)			
Tobacco[c]	0.038					
never vs former	0.021	0.324	(0.124-0.846)	0.058	0.382	(0.141-1.035)
never vs current	0.783	1.139	(0.450-2.883)	0.561	0.742	(0.272-2.027)
Diabetes melitus[c] no/yes	0.374	0.523	(0.125-2.186)			
Cardiovascular disease[c] no/yes	0.062	0.149	(0.020-1.101)	0.110	0.189	(0.024-1.458)
Hypercholesterolemia[c] no/yes	0.419	0.700	(0.294-1.663)			
No. high risk factors (1 base) [c, n]	0.004					
1 vs 2	0.387	1.606	(0.550-4.689)			
1 vs 3	0.001	28.218	(3.822-208.342)			

[a] ADT duration was not included in the multivariate analysis because it is directly related to ADT.

[c] Categorical variable. [g] Gleason group variable was entered as a continuous variable. [n] count are already included in the multivariate analysis.

Supplementary Table S1c. All-cause mortality univariate and multivariate analysis.

	All-Cause Mortality					
	Univariate			Multivariate		
Variable	p	HR	(CI)	p	HR	(CI)
Age	< 0.001	1.065	(1.044-1.085)	< 0.001	1.075	(1.053-1.098)
Pre-implant PSA	0.142	0.992	(0.981-1.003)			
Gleason group[g]	0.019	1.170	(1.021-1.341)	0.163	1.105	(0.960-1.272)
Body mass index	0.749	1.005	(0.976-1.034)			
Percent positive biopsies	0.025	1.005	(1.001-1.010)	0.047	1.005	(1.000-1.010)
Perineural Invasion no/yes	0.794	1.034	(0.802-1.334)			
%D90	0.469	0.996	(0.987-1.006)			
EBRT[c] no/yes	0.256	0.711	(0.406-1.246)			
Post-2004 implant No/yes	0.098	0.781	(0.582-1.047)	0.113	0.780	(0.574-1.060)
ADT[c] no/yes	0.602	1.077	(0.815-1.423)			
ADT duration [a, c]	0.720					
none vs < 6 months	0.413	1.185	(0.789-1.779)			
none vs > 6 months	0.748	1.049	(0.785-1.401)			
Hypertension[c] no/yes	0.463	1.101	(0.852-1.422)			
Tobacco[c]	0.085					
never vs former	0.0429	1.123	(0.842-1.498)	0.293	1.169	(0.874-1.563)
never vs current	0.023	1.537	(1.060-2.230)	< 0.001	2.374	(1.587-3.550)
Diabetes melitus[c] no/yes	0.195	1.269	(0.894-1.803)			
Cardiovascular disease[c] no/yes	0.067	1.340	(0.988-1.816)	0.692	1.073	(0.758-1.520)
Hypercholesterolemia[c] no/yes	0.043	1.323	(1.013-1.730)	0.137	1.264	(0.928-1.721)
No. high risk factors (1 base) [c, n]	0.996					
1 vs 2	0.932	0.981	(0.638-1.510)			
1 vs 3	0.995	1.004	(0.247-4.084)			

[a] ADT duration was not included in the multivariate analysis because it is directly related to ADT.

[c] Categorical variable.[g] Gleason group variable was entered as a continuous variable. [n] count are already included in the multivariate analysis.
